# Assessment of pulmonary vascular volume and lung perfusion in patients with hypoplastic left heart syndrome (HLHS) in Fontan-circulation

**DOI:** 10.1186/1532-429X-13-S1-P198

**Published:** 2011-02-02

**Authors:** Carsten Rickers, Eileen Pardun, Michael Jerosch-Herold, Dominik Gabbert, Chris Hart, Inga Voges, Andreas Entenmann, Hans Heiner Kramer

**Affiliations:** 1 University Hospital Schleswig-Holstein, Kiel, Germany; 2 Brigham and Womens Hospital, Harvard Medical School, Boston, MA, USA

## Introduction

Data from catheter angiography indicate that children with Hypoplastic Left Heart Syndrome (HLHS) in Fontan circulation have hypoplastic central pulmonary arteries. We used novel MRI techniques to quantify pulmonary vascular volume and lung perfusion in a cohort of HLHS patients and a group of healthy control subjects.

## Methods

We investigated 31 children (4,9±2,3 yrs) with HLHS, and 6 lung healthy children (9,8±6,4 yrs). A modified contrast-enhanced dynamic MR-angiography sequence was used to assess pulmonary vascular volume and total lef and right lung volumes. CMR software was used to measure, total lung volume, and the up-slope of the signal intensity curves, as well as the mean transit time (MTT), in order to assess pulmonary perfusion.

## Results

The indexed pulmonary total lung volume and the relative vascular volume in HLHS was significantly reduced compared to the control group (650±102 vs. 945±274 ml/m^2^, p=0.049; 6.5±3.7 vs. 9.,5±2.4 %/m^2^, p=0.03). There were no differences of the right and the left side of the lungs within the two groups. Lung perfusion in children with HLHS was also impaired (MTT: 10.8±2 vs. 6.7±3 s, p=0.004; Up-slope: 4.8±2,6 vs.10.1±5.8 s-1m^2^; p=0,075). There were no differences of the right and the left side of the lungs within the two groups. Additionally, we found a weak correlation between cardiac index (2.78±0,82ml/m2/min) and lung perfusion (r=0.34, p=0.,062). Additionally, we found a correlation between cardiac index (2,78±0,82ml/m^2^/min) and lung perfusion (r=0,54, p<0,05) in those HLHS pts with the fenestration closed.

## Conclusions

Advanced MRI methods enable the assessment of pulmonary perfusion in HLHS patients in Fontan circulation. HLHS patients have a reduced total lung volume, pulmonary vascular volume, and an impaired lung perfusion, compared to controls. The clinical significance of these findings for the Fontan circulation needs to be evaluated by long-term follow-ups.

